# Temporal trends in leisure-time sedentary behavior among adolescents aged 12-15 years from 26 countries in Asia, Africa, and the Americas

**DOI:** 10.1186/s12966-020-01010-w

**Published:** 2020-08-12

**Authors:** Mireia Felez-Nobrega, Lauren B. Raine, Josep Maria Haro, Katrien Wijndaele, Ai Koyanagi

**Affiliations:** 1 Research and Development Unit, Parc Sanitari Sant Joan de Déu, C/ Dr. Antoni Pujadas 42, 08830 Sant Boi de Llobregat, Barcelona, Spain; 2 Department of Psychology, Northeastern University, 125 NI, 360 Huntington Ave, Boston, MA 02115 USA; 3 Centre for Biomedical Research on Mental Health (CIBERSAM), Madrid, Spain; 4 MRC Epidemiology Unit, University of Cambridge, Cambridge, UK; 5 ICREA, Pg. Lluis Companys 23, Barcelona, Spain

**Keywords:** Adolescents, Longitudinal, Cohort, Sitting, Cross-country comparison

## Abstract

**Background:**

Multi-country studies examining trends in sedentary behaviors among adolescents have mainly focused on high-income or Western countries, and almost no data exists for the rest of the world. Thus, this study aims to examine temporal trends in adolescents’ leisure time sedentary behavior (LTSB) employing nationally representative datasets from 26 countries from five WHO-defined geographical regions.

**Methods:**

Data from the Global School-based Student Health Survey 2003–2017 were analyzed in 17,734 adolescents [mean (SD) age: 13.7 (1.0) years; 49.0% boys]. LTSB was self-reported and included all types of sedentary behaviors, excluding time spent at school or doing homework. The prevalence and 95%CI of high LTSB (i.e., ≥3 h/day) was calculated for the overall sample and by sex for each survey. Crude linear trends in high LTSB were assessed by linear regression models. Interaction analyses were conducted to examine differing trends among boys and girls.

**Results:**

Temporal variations in LTSB substantially diverged across countries, with results showing increasing (6/26 countries), decreasing (4/26) and stable trends. The sharpest increases in LTSB occurred in United Arab Emirates, Kuwait, and Thailand. Some countries did not show an increase in LTSB prevalence over time but had very high levels of LTSB (i.e., > 40%) across multiple years. Most countries showed no differences in LTSB trends between boys and girls.

**Conclusions:**

Data from our study may serve as an important platform for policymakers, as well as local and national stakeholders, to establish country-specific and tailored strategies for reducing LTSB.

## Background

Sedentary behavior represents those behaviors undertaken while sitting or lying, with a low level of energy expenditure (i.e., ≤1.5metabolic equivalent units), excluding sleep [1]. This complex set of behaviors occurs within the context of our daily-living (e.g., work, leisure, transportation) and in different modes (e.g., reading, TV time) [1].

Adolescents are the most sedentary of pediatric populations, and importantly, evidence shows that they spend more than half of their after-school period in sedentary pursuits (57%) [2]. Higher levels of sedentary behavior have been related to a wide range of negative health markers in youth including physical, behavioral and psychological outcomes. Specifically, greater time spent in sedentary behavior is related to higher depressive symptoms, unfavorable body composition, cardiovascular risk factors, poor fitness, lower self-esteem, and lower quality of life [3–6]. Importantly, those sedentary behaviors that occur during leisure-time may be the most important, since they have been more consistently associated with health outcomes [4, 6].

The steepest increases in sedentary behaviors may occur during early adolescence (9–12 years) [7], and perhaps more importantly, sedentary behavior in childhood may persist into adulthood [8], which may compromise youth’s present and future health. Nonetheless, temporal trends of sedentary behavior during adolescence at the population level remain poorly described. From an international perspective, multi-country studies examining these trends have mainly focused on high-income or Western countries [9–11]. Similarly, single-country studies examining SB trends have mostly covered the same geographical regions, with the majority of evidence being derived from North American and European cohorts [12–17]. Only a few studies have examined secular changes in non-high-income countries, more specifically in Chinese, Filipino, and Brazilian adolescents [18–21].

While the existing literature is informative, there are a number of limitations that warrant further research. Available data derived solely from a single country cohort do not allow extrapolation of findings beyond the setting where the study was conducted, and the use of discrepant research designs and methodologies makes comparison between studies difficult. Multi-country studies with standardized methods across countries allow for internationally comparable estimates to better monitor trends over time among countries. However, previous multi-country studies have mostly been restricted to Western high-income countries [9–11] and studies from other settings are necessary to provide a better understanding of global sedentary behavior trends. For instance, it is possible that in low- and middle-income countries (LMICs), trends in sedentary behavior may differ from those of high-income countries due to less access to electronic devices, computers, internet or TV, while rapid changes in these behaviors may be occurring due to accelerated urbanization processes, globalization, and changes in lifestyles [22].

Thus, the current study aimed to describe trends in leisure-time sedentary behavior (LSTB) using nationally representative data from 26 countries from five WHO-defined geographical regions (African Region, Region of the Americas, Eastern Mediterranean Region, South-East Asia Region, Western Pacific Region) for which no prior data on temporal trends of sedentary behavior among adolescents exists, with the exception of the Philippines [20]. Adolescence is a highly vulnerable period, with changes in lifestyle and environments underlying the need for a better understanding of trends in sedentary behavior, especially for the development of tailored intervention strategies and public health efforts.

## Methods

Publicly available data from the Global School-based Student Health Survey (GSHS) were analyzed. Details on this survey can be found at http://www.who.int/chp/gshs and http://www.cdc.gov/gshs. Briefly, the GSHS was jointly developed by the WHO and the US Centers for Disease Control and Prevention (CDC), and other UN allies. The core aim of this survey was to assess and quantify risk and protective factors of major non-communicable diseases. The survey draws content from the CDC Youth Risk Behavior Survey (YRBS) for which test-retest reliability has been established [23]. The survey used a standardized two-stage probability sampling design for the selection process within each participating country. For the first stage, schools were selected with probability proportional to size sampling. The second stage involved the random selection of classrooms which included students aged 13–15 years within each selected school. All students in the selected classrooms were eligible to participate in the survey regardless of age. Data collection was performed during one regular class period. The questionnaire was translated into the local language in each country and consisted of multiple choice response options; students recorded their response on computer scannable sheets. All GSHS surveys were approved, in each country, by both a national government administration (most often the Ministry of Health or Education) and an institutional review board or ethics committee. Student privacy was protected through anonymous and voluntary participation, and informed consent was obtained as appropriate from the students, parents and/or school officials. Data were weighted for non-response and probability selection.

From all publicly available data, we selected all nationally representative datasets that included the variables used in the current analysis, and countries for which data on at least two waves were available. Thus, a total of 26 countries were included in the current study. The characteristics of each country or survey are provided in Table 1. For the included countries, the survey was conducted between 2003 and 2017.

**Table 1:** Survey characteristics

Region	Country	Country income	Year	Response rate (%)	N^a^
AFR	Benin	L	2009	90	1170
		L	2016	78	717
	Mauritius	UM	2007	88	1961
		UM	2011	82	2074
		UM	2017	84	1955
	Namibia	LM	2004	82	4529
		UM	2013	89	1936
	Seychelles	UM	2007	82	1154
		H	2015	82	2061
	Argentina	UM	2007	77	1537
		UM	2012	71	21,528
	Guatemala	LM	2009	81	4495
		LM	2015	82	3611
	Guyana	LM	2004	80	1070
		LM	2010	76	1973
	Suriname	UM	2009	89	1046
		UM	2016	83	1453
	Trinidad & Tobago	H	2007	78	2450
		H	2011	90	2363
		H	2017	89	2763
	Uruguay	UM	2006	71	2882
		H	2012	77	2869
EMR	Egypt	LM	2006	87	4981
		LM	2011	85	2364
	Jordan	LM	2004	95	1848
		LM	2007	99.8	1648
	Kuwait	H	2011	85	2298
		H	2015	78	2034
	Lebanon	UM	2011	87	1982
		UM	2017	82	3347
	Morocco	LM	2006	84	1986
		LM	2010	92	2405
		LM	2016	91	3975
	Oman	UM	2005	97	2426
		H	2010	89	1000
		H	2015	92	1669
	United Arab Emirates	H	2005	89	12,819
		H	2010	91	2302
		H	2016	80	3471
	Yemen	L	2008	82	905
		LM	2014	75	1553
SEAR	Indonesia	LM	2007	93	3022
		LM	2015	94	8806
	Myanmar	L	2007	95	2227
		LM	2016	86	2237
	Sri Lanka	LM	2008	89	2504
		LM	2016	89	2254
	Thailand	LM	2008	93	2675
		UM	2015	89	4132
WPR	Fiji	LM	2010	90	1495
		UM	2016	79	1537
	Philippines	LM	2003	84	4198
		LM	2007	81	3484
		LM	2011	82	3845
		LM	2015	79	6162
	Tonga	LM	2010	80	1946
		UM	2017	90	2067
	Vanuatu	LM	2011	72	852
		LM	2016	57	1288

*Abbreviation*: *AFR* African Region, *AMR* Region of the Americas, *EMR* Eastern Mediterranean Region, *SEAR* South-East Asia Region, *WPR* Western Pacific Region, *H* high income, *L* low incomem *LM* lower middle-income, *UM* upper middle-income. Income classification is based on the World Bank classification at the time of the survey

^a^Based on sample aged 12–15 years

### Leisure-time sedentary behavior (LSTB)

LTSB was assessed with the question “How much time do you spend during a typical or usual day sitting and watching television, playing computer games, talking with friends, or doing other sitting activities?” with answer options: < 1, 1–2, 3–4, 5–6, 7–8, and ≥ 8 h/day. This excluded time at school and when doing homework. In accordance with previous research showing that engaging in sedentary behavior for ≥3 h/day is associated with significant health risks [5, 24–28], the variable was dichotomized as ≥3 h/day (high LTSB) or not.

### Statistical analysis

Statistical analyses were performed with Stata 14.1 (Stata Corp LP, College station, Texas). The analysis was restricted to those aged 12–15 years as most students were within this age group while information on the exact age outside of this age range was not available. The prevalence and 95%CI of high LTSB (i.e., ≥3 h/day) was calculated for the overall sample and by sex for each survey year and for all the included countries. Crude linear trends in high LTSB were assessed by linear regression models across surveys within the same country to estimate regression coefficients (beta) and 95%CI for every one-year change. P for trends were estimated using the survey year as a continuous variable. We also conducted interaction analysis to assess whether there are differing trends among boys and girls by including an interaction term (survey year X sex) in the model. Sampling weights and the clustered sampling design of the surveys were taken into account in all analyses.

## Results

**Fig. 1:**
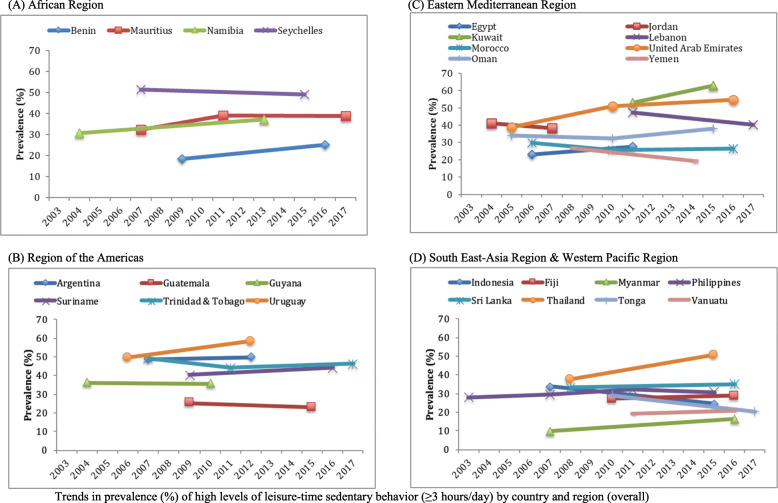
Trends in prevalence (%) of high levels of leisure-time sedentary behavior (≥3 h/day) by country and region (overall). **a** African Region. **b** Region of the Americas. **c** Eastern Mediterranean Region. **d** South East-Asia Region & Western Pacific Region

**Fig. 2:**
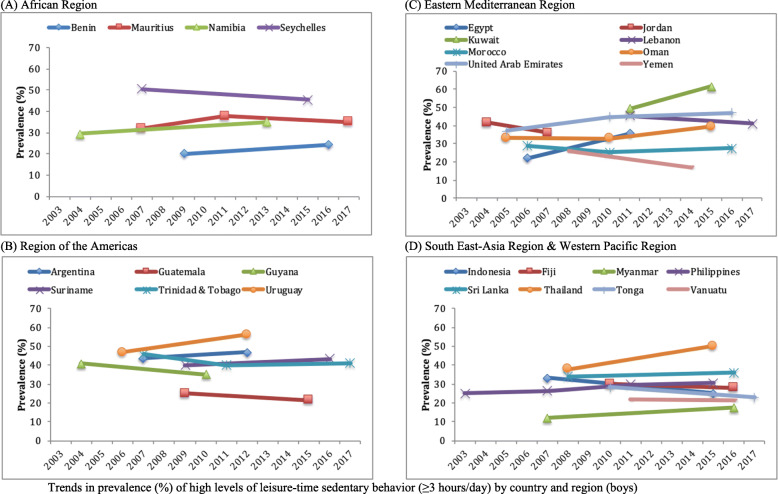
Trends in prevalence (%) of high levels of leisure-time sedentary behavior (≥3 h/day) by country and region (boys). **a** African Region. **b** Region of the Americas. **c** Eastern Mediterranean Region. **d** South East-Asia Region & Western Pacific Region

**Fig. 3:**
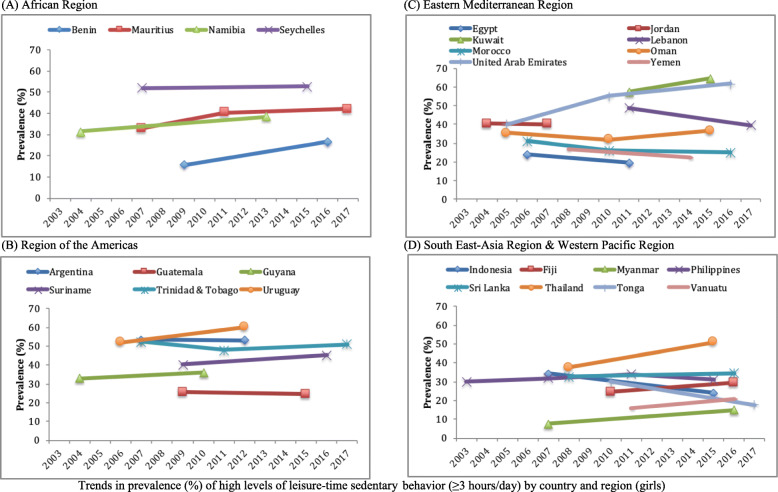
Trends in prevalence (%) of high levels of leisure-time sedentary behavior (≥3 h/day) by country and region (girls). **a** African Region. **b** Region of the Americas. **c** Eastern Mediterranean Region. **d** South East-Asia Region & Western Pacific Region

Data on 17,734 students aged 12–15 years [mean (SD) age 13.7 (1.0) years; 49.0% boys] were used for the current analysis. The trends in sedentary behavior are shown in Table 2, Fig. 1 (overall), Fig. 2 (boys), and Fig. 3 (girls). Overall, the prevalence of high LTSB (i.e., ≥3 h/day) ranged from 9.7% in Myanmar (2007) to 62.9% in Kuwait (2015). Overall, significant increasing trends for high LTSB were observed in Namibia between 2004 (30.7%) and 2013 (37.2%) (beta = 0.71; 95%CI = 0.35,1.08), Uruguay between 2006 (49.6%) and 2012 (58.3%) (beta = 1.45; 95%CI = 0.82,2.08), Kuwait between 2011 (53.1%) and 2015 (62.9%) (beta = 2.45; 95%CI = 0.56,4.34), United Arab Emirates between 2005 (38.8%) and 2016 (54.7%) (beta = 1.41; 95%CI = 1.01,1.81), Myanmar between 2007 (9.7%) and 2016 (16.2%) (beta = 0.73; 95%CI = 0.35,1.10), and Thailand between 2008 (37.6%) and 2015 (50.7%) (beta = 1.86; 95%CI = 1.08,2.64). The beta can be interpreted as the average percentage point change in prevalence per year. On the other hand, significant decreasing trends were observed in Lebanon between 2011 (47.2%) and 2017 (40.2%) (beta = - 1.17; 95%CI = -2.07,-0.28), Yemen between 2008 (26.8%) and 2014 (19.4%) (beta = - 1.23; 95%CI = -2.24,-0.22), Tonga between 2010 (29.2%) and 2017 (20.3%) (beta = - 1.28; 95%CI = -1.78,-0.78), and Indonesia between 2007 (33.8%) and 2015 (24.5%) (beta = - 1.17; 95%CI = -1.83, -0.51). The overall prevalence of LTSB was stable over time in all other countries.

**Table 2:** Trends in prevalence (%) of high levels of leisure-time sedentary behavior (≥3 h/day) in 26 countries (overall and by sex)

		Overall			Boys			Girls		
Country	Year	% [95%CI]	beta^a^ [95%CI]	p for trend^a^	% [95%CI]	beta^a^ [95%CI]	p for trend^a^	% [95%CI]	beta^a^ [95%CI]	p for trend^a^
**AFR**										
Benin	2009	18.4 [15.7,21.4]	0.96 [−0.01,1.94]	0.052	20.0 [16.8,23.5]	0.62 [−0.52,1.77]	0.276	15.8 [12.3,20.1]	1.55 [0.58,2.51]	0.003
	2016	25.2 [19.7,31.5]			24.3 [18.0,32.0]			26.7 [21.8,32.2]		
Mauritius	2007	32.4 [28.5,36.7]	0.58 [−0.02,1.18]	0.057	31.8 [27.2,36.8]	0.25 [− 0.45,0.94]	0.477	33.0 [26.6,40.0]	0.84 [− 0.14,1.82]	0.090
	2011	39.2 [35.9,42.6]			37.7 [32.7,42.9]			40.5 [36.4,44.8]		
	2017	39.0 [35.0,43.3]			35.1 [30.9,39.7]			42.1 [35.6,48.9]		
Namibia	2004	30.7 [28.8,32.8]	0.71 [0.35,1.08]	< 0.001	29.6 [26.8,32.7]	0.61 [0.07,1.15]	0.028	31.4 [29.0,34.0]	0.78 [0.39,1.17]	< 0.001
	2013	37.2 [34.7,39.7]			35.1 [31.5,39.0]			38.4 [36.1,40.8]		
Seychelles	2007	51.4 [50.7,52.1]	−0.27 [−0.65,0.10]	0.155	50.5 [49.4,51.6]	− 0.64 [−1.15,-0.13]	0.014	52.0 [50.5,53.5]	0.10 [− 0.38,0.58]	0.689
	2015	49.2 [46.3,52.2]			45.4 [41.5,49.3]			52.8 [49.2,56.3]		
**AMR**										
Argentina	2007	48.7 [41.5,56.0]	0.23 [−1.27,1.73]	0.766	43.6 [37.1,50.3]	0.62 [−0.81,2.05]	0.395	53.4 [44.3,62.3]	−0.09 [− 1.96,1.79]	0.928
	2012	49.9 [48.3,51.4]			46.7 [44.0,49.4]			53.0 [50.8,55.1]		
Guatemala	2009	25.2 [21.4,29.5]	−0.39 [− 1.54,0.76]	0.503	25.1 [21.8,28.7]	−0.60 [− 1.78,0.57]	0.313	25.6 [20.7,31.1]	−0.17 [−1.51,1.16]	0.797
	2015	22.9 [17.8,28.8]			21.5 [16.0,28.2]			24.5 [19.1,30.9]		
Guyana	2004	36.3 [32.5,40.3]	−0.11 [−1.19,0.98]	0.844	40.8 [34.9,47.0]	−0.96 [−2.32,0.40]	0.159	32.8 [28.1,37.8]	0.54 [−0.80,1.89]	0.416
	2010	35.7 [30.9,40.7]			35.0 [30.3,40.1]			36.0 [30.2,42.2]		
Suriname	2009	40.3 [35.8,44.9]	0.57 [−0.25,1.39]	0.166	40.0 [33.9,46.5]	0.47 [−0.61,1.55]	0.377	40.3 [36.0,44.8]	0.69 [−0.18,1.57]	0.116
	2016	44.2 [41.2,47.3]			43.4 [39.9,46.9]			45.2 [41.3,49.1]		
Trinidad & Tobago	2007	49.1 [46.2,52.1]	−0.26 [−0.68,0.15]	0.216	45.6 [41.5,49.7]	−0.43 [−1.04,0.19]	0.171	52.3 [48.1,56.6]	−0.12 [− 0.71,0.46]	0.678
	2011	44.0 [40.7,47.4]			40.1 [33.6,46.8]			48.0 [44.8,51.2]		
	2017	46.2 [43.3,49.1]			41.0 [36.4,45.8]			50.8 [46.7,54.8]		
Uruguay	2006	49.6 [47.3,51.9]	1.45 [0.82,2.08]	< 0.001	46.8 [43.1,50.6]	1.56 [0.69,2.43]	0.001	51.9 [49.3,54.5]	1.37 [0.59,2.15]	0.001
	2012	58.3 [55.4,61.2]			56.2 [52.7,59.6]			60.1 [56.3,63.8]		
**EMR**										
Egypt	2006	23.1 [18.6,28.3]	0.88 [−0.60,2.37]	0.239	22.0 [18.1,26.4]	2.72 [1.00,4.44]	0.002	24.0 [16.8,33.0]	−0.87 [−2.85,1.10]	0.377
	2011	27.5 [22.5,33.3]			35.6 [28.7,43.2]			19.6 [15.0,25.3]		
Jordan	2004	41.2 [38.0,44.6]	−1.01 [−2.85,0.82]	0.267	41.7 [36.5,47.2]	−1.91 [−4.39,0.57]	0.125	40.5 [37.0,44.2]	− 0.16 [−2.74,2.43]	0.901
	2007	38.2 [34.2,42.3]			36.0 [31.5,40.7]			40.1 [33.8,46.7]		
Kuwait	2011	53.1 [48.5,57.7]	2.45 [0.56,4.34]	0.013	49.2 [41.8,56.6]	3.04 [0.40,5.69]	0.026	57.3 [52.3,62.2]	1.78 [−0.35,3.91]	0.097
	2015	62.9 [57.1,68.3]			61.4 [54.3,67.9]			64.5 [57.9,70.6]		
Lebanon	2011	47.2 [42.9,51.6]	−1.17 [−2.07,-0.28]	0.011	45.3 [40.1,50.7]	−0.72 [− 1.78,0.35]	0.182	48.9 [44.7,53.2]	−1.57 [− 2.51,-0.63]	0.002
	2017	40.2 [37.4,43.1]			41.0 [37.8,44.3]			39.5 [36.1,43.0]		
Morocco	2006	29.9 [27.6,32.4]	−0.30 [−0.71,0.11]	0.147	29.0 [26.0,32.3]	−0.08 [− 0.56,0.41]	0.755	31.1 [27.6,34.9]	−0.55 [− 1.05,-0.05]	0.033
	2010	25.7 [23.2,28.4]			25.3 [22.6,28.3]			26.0 [22.7,29.6]		
	2016	26.3 [23.3,29.5]			27.6 [24.2,31.3]			25.0 [21.8,28.5]		
Oman	2005	34.2 [31.0,37.5]	0.33 [−0.16,0.83]	0.181	33.2 [30.0,36.5]	0.57 [0.00,1.14]	0.050	35.3 [29.3,41.8]	0.07 [− 0.80,0.93]	0.875
	2010	32.4 [29.0,36.0]			32.9 [29.6,36.5]			31.8 [26.7,37.4]		
	2015	38.0 [34.4,41.6]			39.4 [34.7,44.3]			36.4 [31.0,42.2]		
United Arab Emirates	2005	38.8 [37.1,40.4]	1.41 [1.01,1.81]	< 0.001	37.2 [35.3,39.2]	0.88 [0.44,1.33]	< 0.001	40.2 [37.9,42.5]	1.90 [1.42,2.39]	< 0.001
	2010	51.0 [47.1,54.8]			44.8 [40.2,49.4]			55.1 [50.2,59.8]		
	2016	54.7 [50.7,58.6]			47.2 [43.0,51.4]			61.7 [57.0,66.1]		
Yemen	2008	26.8 [23.1,30.8]	−1.23 [−2.24,-0.22]	0.018	26.2 [22.6,30.3]	−1.56 [−2.71,-0.41]	0.009	26.7 [20.8,33.6]	−0.73 [− 2.15,0.68]	0.297
	2014	19.4 [15.4,24.0]			16.9 [12.2,22.9]			22.3 [17.8,27.6]		
**SEAR**										
Indonesia	2007	33.8 [29.2,38.8]	−1.17 [−1.83,-0.51]	0.001	33.1 [27.8,38.9]	−1.01 [− 1.78,-0.25]	0.010	34.4 [29.5,39.7]	−1.31 [−2.02,-0.61]	< 0.001
	2015	24.5 [22.8,26.3]			25.0 [22.9,27.3]			23.9 [21.9,26.0]		
Myanmar	2007	9.7 [7.6,12.2]	0.73 [0.35,1.10]	< 0.001	11.9 [9.2,15.3]	0.60 [0.11,1.09]	0.018	7.5 [5.6,9.8]	0.84 [0.47,1.21]	< 0.001
	2016	16.2 [13.9,18.7]			17.3 [14.5,20.6]			15.0 [12.7,17.7]		
Sri Lanka	2008	33.2 [31.0,35.4]	0.25 [−0.41,0.90]	0.449	33.9 [30.5,37.4]	0.26 [−0.58,1.11]	0.535	32.6 [30.0,35.3]	0.24 [−0.55,1.03]	0.542
	2016	35.1 [30.7,39.8]			36.0 [30.6,41.7]			34.5 [29.2,40.2]		
Thailand	2008	37.6 [33.2,42.3]	1.86 [1.08,2.64]	< 0.001	37.7 [33.4,42.2]	1.79 [0.87,2.71]	< 0.001	37.5 [31.6,43.8]	1.92 [0.86,2.98]	0.001
	2015	50.7 [47.9,53.4]			50.2 [45.8,54.7]			50.9 [47.1,54.8]		
**WPR**										
Fiji	2010	27.2 [23.5,31.2]	0.28 [−0.62,1.19]	0.528	29.9 [24.3,36.2]	−0.34 [−1.52,0.84]	0.560	24.6 [18.8,31.5]	0.78 [−0.56,2.12]	0.245
	2016	28.9 [25.5,32.5]			27.9 [24.7,31.3]			29.3 [25.1,33.8]		
Philippines	2003	27.9 [23.9,32.5]	0.22 [−0.25,0.69]	0.353	25.1 [20.0,30.9]	0.46 [−0.15,1.07]	0.139	29.9 [25.8,34.5]	0.07 [−0.44,0.58]	0.782
	2007	29.5 [24.5,35.0]			26.4 [22.1,31.3]			31.9 [25.8,38.6]		
	2011	32.0 [25.8,38.9]			29.7 [23.4,36.8]			34.1 [27.4,41.5]		
	2015	30.7 [27.1,34.5]			30.4 [25.4,35.8]			31.0 [27.0,35.2]		
Tonga	2010	29.2 [26.6,32.0]	−1.28 [−1.78,-0.78]	< 0.001	28.4 [25.0,32.1]	−0.81 [− 1.48,-0.14]	0.019	30.0 [26.5,33.7]	−1.75 [−2.38,-1.11]	< 0.001
	2017	20.3 [18.2,22.6]			22.8 [25.0,32.1]			17.7 [15.3,20.4]		
Vanuatu	2011	19.0 [14.5,24.5]	0.40 [−0.79,1.58]	0.505	22.1 [15.0,31.3]	−0.11 [−1.92,1.70]	0.900	15.8 [11.7,21.0]	0.98 [−0.28,2.25]	0.126
	2016	21.0 [18.2,24.0]			21.5 [18.3,25.1]			20.7 [16.9,25.1]		

*Abbreviation*: *CI* Confidence interval, *AFR* African Region, *AMR* Region of the Americas, *EMR* Eastern Mediterranean Region, *SEAR* South-East Asia Region, *WPR* Western Pacific Region

^a^The beta and P for trend are based on linear regression including survey year as a continuous variable. The beta can be interpreted as the average percentage point change in prevalence per year

Most countries showed similar trends between boys and girls, but interaction analysis showed that there were significant differences in trends by sex in Seychelles, Egypt, United Arab Emirates, and Tonga. In Seychelles, a significant decreasing trend was only observed among boys (b = - 0.64; 95%CI = -1.15,-0.13) between 2007 (50.5%) and 2015 (45.4%), while in Egypt, a significant increasing trend was only observed among boys (b = 2.72; 95%CI = 1.00,4.44) between 2006 (22.0%) and 2011 (35.6%). The significant increasing trend observed in United Arab Emirates between 2005 and 2016, and the significant decreasing trend in Tonga between 2010 and 2017 were both driven mainly by the more pronounced trend among girls.

## Discussion

This multicounty study that examined temporal trends in adolescents’ LTSB in a large population sample of 26 under-represented countries from five WHO-defined geographical regions allowed for the extension of previous research by estimating LTSB over time among adolescents in world regions where almost no data exists.

The current study found that temporal variations in LTSB substantially diverged across countries, with results showing increasing, decreasing and stable trends. Furthermore, concerningly high rates of LTSB were found for several countries. Former evidence tends to show increasing trends over time independently of method of measurement or type of sedentary behavior. For instance, single country studies found that self-reported out-of-school screen time (including or not including homework hours), increased over time in adolescents from Hong Kong, UK, Estonia and Czech Republic, and mainland China [14–16, 18, 19, 29]. Additionally, a recent US study specifically showed stable prevalence for watching TV/videos and significant increases in adolescents’ computer use between 2001 and 2016 [12]. Similar findings were shown in a LMIC setting where Brazilian adolescents spent less time watching TV between 2001 and 2011, whereas leisure computer use increased during the same period [21]. Beyond leisure screen time, other leisure-based sedentary behaviors (e.g., reading, writing) and time spent sedentary doing homework increased in adolescents from Mainland China and Hong Kong at different time points between 1997 and 2006 [18] and between 1995 and 2000 [29], although there were exceptions (e.g., decreasing trends in homework-related sedentary behavior among adolescents of mainland China between 2004 and 2011 [19]). Next, total sedentary time assessed via accelerometry (2013–2015) or via self-report (2007–2016) increased over time in Finnish and US adolescents, respectively [12, 13]. Two large multi-country studies mostly based in Western or high-income countries found that adolescents’ TV viewing time decreased, while computer use (including leisure and non-leisure purposes) sharply increased over time in most included countries between 2002 and 2014 [9, 10]. Similarly, increases in total accelerometry-derived sedentary behavior (harmonized datasets from 1997 to 2014) were found in a study including adolescents from several European countries [11].

Differences in research designs and methodologies regarding the assessment of different constructs and dimensions of sedentary behavior hinder comparability between our results and those from these previous studies. That is, while the present study relied on a more comprehensive definition of sedentary behavior that included all types of LTSB (i.e., screen and non-screen based), the vast majority of previous studies have assessed either TV time and computer use (including leisure and non-leisure purposes such as homework) solely or total self-reported/accelerometer-measured sedentary behavior. The only study that used a comparable measure of sedentary behavior with ours (i.e., a composite measure of LTSB including screen and non-screen time) found that trends remained stable over time in the Philippines [20], and this concurs with our findings from this country.

The present study revealed increasing trends in LTSB in several countries across multiple regions. The sharpest increases in LTSB occurred in United Arab Emirates, Kuwait, and Thailand, countries where more than half of the adolescents (50.7–62.9%) reported engagement in high LTSB (≥3 h/day) based on most recent data. Sedentary behavior is a complex behavior that is influenced by multiple determinants. In high-income countries, advances in digital technologies have created an environment that promotes higher LTSB [30], and LMICs are also following this trend owing to the rapid spread of digital technologies (e.g., internet coverage, mobile infrastructure, smartphone connections) in recent years [31, 32]. For instance, it has been estimated that 3G coverage increased from around 60% in 2014 to more than 90% in 2018 in Indonesia [32]. Thus, the rapid increase in access to TV, computer games and overall new technologies may have contributed to the increasing trend in LTSB observed in our study. Furthermore, globalization, urbanization and changes in built environments may also be contributing to the rising trend of LTSB especially in LMICs. In some regions, modern facilities and electricity contributed to replacing physically active chores (e.g., fetching water, firewood, gardening, cattle herding) for greater time spent in LTSB such as studying, watching TV and listening to the radio [33, 34].

While several national and international guidelines for adolescents recommend to break prolonged sedentary behavior and to limit recreational screen time [35–37], to our knowledge, no large-scale intervention studies to reduce sedentary behavior have been conducted to date. Following the seminal intervention study conducted two decades ago that aimed to reduce leisure screen-time in American elementary school children [38], previous evidence, mostly derived from high-income countries, have found that interventions aimed at reducing sedentary behavior in young people show small but statistically significant effects [39]. Most promising strategies to reduce sedentary behavior were multi-component strategies including behavioral (e.g., focused on theory driven approaches), and environmental (e.g., aim to modify home, school, or facilities) approaches, interventions that were conducted in community settings, and those that included the involvement of family members [39, 40]. More research regarding key features or effective interventions for reducing adolescent’s sedentary behavior is needed from more culturally diverse settings including LMICs. In our study, significant decreasing trends in LTSB over time were found in Lebanon, Yemen, and Tonga, and future research that aim to understand the reasons why LTSB is decreasing in some settings may provide clues on how to establish effective public health interventions to reduce sedentary behavior among adolescents.

The examination of trends in sedentary behavior by sex is crucial to determine whether behavior patterns and intervention needs are gender specific. The present study revealed that most countries showed no differences in LTSB trends between boys and girls. The evidence on sex-difference and sedentary behavior prevalence is still limited and sedentary behavior type-dependent. Data from single point multi-county studies showed that accelerometry-measured total sedentary time is higher in European girls than boys [41], while another cross-national study based in Latin America and the Caribbean countries found that girls reported higher LTSB in all the eleven countries where sex-differences in LTSB were found [42]. Nonetheless, despite baseline differences, changes over time seem to occur in similar directions for boys and girls [9, 10, 43], which is what was observed in most of the countries included in our study. However, in the current study, differences in LTSB over time between boys and girls were apparent in some countries, with the most pronounced contrast in terms of sex differences being observed in Egypt and United Arab Emirates. Specifically, boys in Egypt, and girls in United Arab Emirates showed more abrupt increases in LTSB over time compared to the opposite sex. Although the underlying reasons for these findings can only be speculated, in some Muslim-majority countries, restrictions on mobility, less access to some public spaces and less public life, factors that occur in girls’ adolescence [44], may render them more sedentary. On the other hand, adolescent boys may be more likely to engage in LTSB by attending social gatherings outside of home [45]. These cultural factors may have become more widespread in some settings over the past years, but clearly, more research is needed to understand why there are gender differences in LTSB trends in some countries.

The strengths of the study include the large sample size and use of standardized methodology across countries which allowed direct comparisons between countries. Furthermore, we report temporal trends on LTSB from numerous countries spanning multiple continents where data were mostly non-existent. However, the study results should be interpreted in the light of several limitations. First, self-reported estimates of sedentary behavior have well-documented limitations [46]. Although large multi-country studies employing accelerometry-based measures are starting to emerge [11], the use of these tools for estimation of total sedentary behavior is currently limited to certain world regions. In addition, accelerometry measures do not provide accurate estimates of sedentary behavior, and device-based measures are unable to identify daily-living domains and modes of sedentary behavior [46–48], which may be crucial to gain insights into potential targets for intervention strategies. Hence, while methodological approaches continue to improve, self-reported measures used in our study allow direct cross-country comparisons across five different WHO-defined world regions. Another limitation of the current study is that data were restricted to those adolescents attending school, and thus, the results may not be generalizable to adolescents who do not attend school. Relatedly, it is possible that the characteristics of students that attend school have changed over the years and this may have influenced the temporal trends observed in our study. Next, surveys were conducted in different years depending on the country and thus, estimates such as average percentage point change are not totally comparable across countries. Finally, given that some countries provided more data points than others, it is possible that the temporal trends are more accurate in some countries than others.

In conclusion, this study showed that temporal variations in adolescents’ LTSB do not show a consistent trend across all geographic areas, with trends increasing in six of 26 countries, decreasing in four countries, and remaining stable for the rest. Sex differences were observed only in a few countries. Some countries (e.g., Seychelles, Argentina, Suriname Trinidad & Tobago) did not show an increase in LTSB prevalence over time but had very high levels of LTSB (i.e., > 40%) across multiple years, highlighting the need to intervene even in countries where trends are stable. Data from our study may serve as an important platform for policymakers, as well as local and national stakeholders to establish country-specific and tailored strategies for reducing LTSB and the associated negative health outcomes in adolescents.

## Data Availability

The dataset supporting the conclusions of this article is available in: http://www.who.int/chp/gshs

## Abbreviations

LTSB: Leisure time sedentary behavior
LMICs: Low- and middle-income countries
GSHS: Global School-based Student Health Survey
CDC: Centers for Disease Control and Prevention
YRBS: Youth Risk Behavior Survey
